# Lipocalin Blc is a potential heme-binding protein

**DOI:** 10.1002/1873-3468.14001

**Published:** 2020-12-03

**Authors:** Nina G. Bozhanova, M. Wade Calcutt, William N. Beavers, Benjamin P. Brown, Eric P. Skaar, Jens Meiler

**Affiliations:** 1 Department of Chemistry, Center for Structural Biology, Vanderbilt University, Nashville, TN, USA; 2 Mass Spectrometry Research Center, Department of Biochemistry, Vanderbilt University School of Medicine, Nashville, TN, USA; 3 Department of Pathology, Microbiology, and Immunology, Vanderbilt University Medical Center, Nashville, TN, USA; 4 Institute for Drug Discovery, Medical School, Leipzig University, Germany

**Keywords:** Blc, crystal structure, hematoporphyrin, heme-binding protein, lipocalins

## Abstract

Lipocalins are a superfamily of functionally diverse proteins defined by a well-conserved tertiary structure despite variation in sequence. Lipocalins bind and transport small hydrophobic molecules in organisms of all kingdoms. However, there is still uncertainty regarding the function of some members of the family, including bacterial lipocalin Blc from *Escherichia coli*. Here, we present evidence that lipocalin Blc may be involved in heme binding, trans-periplasmic transport, or heme storage. This conclusion is supported by a cocrystal structure, mass-spectrometric data, absorption titration, and *in silico* analysis. Binding of heme is observed at low micromolar range with one-to-one ligand-to-protein stoichiometry. However, the absence of classical coordination to the iron atom leaves the possibility that the primary ligand of Blc is another tetrapyrrole.

Heme is an essential molecule for aerobic organisms. Heme-containing proteins are crucial for many vital biological processes. This includes classical oxygen storage and transport, electron transfer, and catalysis, as well as more recently discovered roles in transcriptional gene regulation and ion channel regulation, among others [1].

Lipocalins are a superfamily of functionally diverse proteins with remarkably conserved tertiary structure despite significant sequence variation. These ~ 20 kDa proteins form an eight-stranded antiparallel β-barrel enclosing an internal ligand-binding pocket. Lipocalins bind and transport small hydrophobic molecules in organisms of all three kingdoms of life. These include, but are not restricted to, fatty acids [2], retinoic acid and retinol [3], heme [4,5], and open tetrapyrroles [6–8].

Heme-binding lipocalins include members from nitrophorins family and α_1_-microglobulin. Nitrophorins are salivary proteins found in several blood-sucking arthropods. Nitrophorins use their prosthetic heme molecule to bind and transport nitric oxide (NO). NO induces vasodilation when released in a host organism upon biting. Nitrophorins are also shown to bind histamine, reducing inflammation and immune response elicited by the insects’ salivary proteins [9]. α_1_-microglobulin is a small globular protein found in a number of vertebrate species. It is known to play a role in clearing cytosols and extravascular fluids of heme groups and free radicals released from hemoglobin [10]. Contrary to classical α-helical globins with deeply buried ferrous heme, heme-binding lipocalins have shallow binding pockets. This leads to high solvent exposure of the heme and allows only for its binding in the ferric form. These differences in structural organization and heme redox properties cause functional diversity between these groups of proteins [11].

The precise biological function of some lipocalin family proteins is not yet clear, even for those discovered more than two decades ago. This includes the first bacterial lipocalin protein identified, *Escherichia coli* lipocalin Blc. Based on monitoring of β-galactosidase activity from a *blc::lacZ* translational fusion reporter plasmid, it was proposed that Blc is involved in cell stress response (starvation, high osmolarity) [12,13]. Later, ligand-binding studies using tryptophan fluorescence quenching proposed that Blc plays a role in the metabolism of lysophospholipids based on its nanomolar affinity for oleoyl-*sn*-2-lysophosphatidylethanolamine and palmitoyl-*sn*-2-lyso-phosphatidylethanolamine [14]. That study, as well as the previous structural analysis of Blc in *apo* state [15], suggests that the protein exists as a functional homodimer. However, biochemical and structural characterization of Blc expressed using a different construct design questions the formation of the homodimer and, correspondingly, the correctness of the previous findings [16]. Recently, an *E. coli* single-gene knockout library screening showed involvement of Blc in the regulation of host intestine immune response against microbes [17]. Particularly, the complete absence of the protein or the mutation of glycine to glutamic acid at position 84 of the protein (a mutation frequently observed in pathogenic *E. coli* strains) is reported to cause activation of local inflammation in the host gut. The G84E Blc mutation has been associated with the higher *E. coli* abundance in the gut and the decrease in lysophosphatidylethanolamine in both the bacteria and the host intestine lumen.

Here, based on a Blc crystal structure and supported by mass-spectrometric data, titration analysis, and molecular dynamics (MD) simulations, we present evidence that lipocalin Blc may also be involved in ferric heme binding, transport, or storage. The absence of the classical proximal coordination of the heme-Fe atom and relatively low binding affinity for heme (*K*_d_ = 1.4 μM) suggests that the Blc protein may not be specific to heme and could participate in handling of other metal-containing or metal-free tetrapyrroles.

## Materials and methods

### Molecular cloning

The vectors containing wild-type Blc (wtBlc)-split-Zip and wtBlc-split (split fragments without leucine zipper peptides) were created using plasmids pMRBad-Z-CspGFP (Addgene, Watertown, MA, USA plasmid #40730), pET11a-Z-NspGFP (Addgene plasmid #40729) [18], which were a gift from Brian McNaughton, and wtBlc [19] as described before [20]. Correctness of the obtained constructs was confirmed by sequencing.

### Protein expression and purification

All proteins were expressed in XJb(DE3) Autolysis (Zymo Research, Irvine, CA, USA) *E. coli* strain. Cells were grown in LB or M9 media supplemented with 100 μg·mL^−1^ ampicillin (full-length wtBlc protein) or 100 μg·mL^−1^ ampicillin and 50 μg·mL^−1^ kanamycin (wtBlc-split-Zip and wtBlc-split proteins) at 37 °C. Expression was induced by addition of 0.04% L-arabinose (full-length wtBlc protein) or 0.2% L-arabinose and 10 μM IPTG (wtBlc-split-Zip and wtBlc-split proteins) at 0.8 OD. Cells were harvested after 3 h of expression at 37 °C if grown in LB or after overnight expression if grown in M9 and were resuspended in PBS buffer, pH 7.4. Suspensions were frozen at −80 °C and thawed at room temperature three times. DNA was destroyed by short sonication, and the lysates were centrifuged to obtain cell-free extracts. The proteins were first purified using gravity flow columns with TALON metal affinity resin (Clontech, Mountain View, CA, USA) and further purified by size-exclusion chromatography on a HiLoad 16/600 Superdex 75 pg or Superdex 200 pg 10/300 GL column (GE Healthcare, Marlborough, MA, USA) pre-equilibrated with 50 mM sodium phosphate buffer, pH 6.0.

### Protein concentration calculation

Protein concentrations were estimated using the Bradford dye-binding method-based [21] colorimetric assay (Bio-Rad, Hercules, CA, USA) and bovine serum albumin standard. Single point absorption measurements (595 nm) were performed using a FlexStation 3 microplate reader (Molecular Devices, San Jose, CA, USA). All measurements were performed in triplicate.

### HPLC-high-resolution MS and MS/MS

Mass-spectrometric experiments were performed using an Acquity UPLC system (Waters, Milford MA, USA) and an LTQ Orbitrap mass spectrometer equipped with a standard electrospray ionization source (Thermo, San Jose, CA, USA). The Acquity system was equipped with a binary solvent manager, refrigerated sample manager, and a thermostated column heater. Samples were chromatographically resolved on a Waters Symmetry300 reverse phase column (C_18_, 2.0 × 100 mm, 3.5 μm) using the following gradient conditions: 0–1 min, B = 0%; 1–8 min, B = 0–100%; 8–10 min, B = 100%; 10–10.5 min, B = 100–0%; 10.5–15 min, B = 0%. The injection volume for all samples was 10 μL. The autosampler injection valve and syringe needle were flushed (1 mL) and rinsed (1 mL) sequentially with mobile phases B and A between each injection. The mass spectrometer was operated in positive ionization mode; the electrospray source parameters were as follows: N_2_ sheath gas, 40; N_2_ aux gas, 5; spray voltage, 4.0 kV; capillary temp, 300 °C; tube lens voltage, 130 V at *m/z* = 1150; capillary voltage, 31 V; skimmer offset, 10 V. Full scan spectra of 300–2000 *m/z* were acquired in FTMS mode at a resolving power of 30 000 FWHM with the following AGC parameters: FT target, 1e6; one microscan, maximum inject time, 50 ms. Collision-induced dissociation of the precursor ion 616.17 *m/z* was performed in the linear ion trap using the following activation parameters: IT target, 1e4; one microscan, maximum inject time, 25 ms; activ Q, 0.25; activ time 30 ms; norm collision energy 15%; isolation width 2.0 *m/z*. Data acquisition and analysis were carried out using XCALIBUR v. 2.0.7 (Thermo Fisher Scientific, Waltham, MA, USA).

### Spectrophotometric hemin titration

A fresh 2 mM hemin stock solution was prepared by adding 5 mL 0.1 M NaOH to 6.5 mg hemin chloride (Sigma-Aldrich, Darmstadt, Germany). The required amount of hemin stock solution (0–6 μL) supplemented with 0.1 M NaOH to a final volume of 6 μL was mixed either with 994 μL of the 50 mM sodium phosphate buffer, pH 6.0 or with the appropriate amounts of the full-length wtBlc or the wtBlc-split protein and 50 mM sodium phosphate buffer, pH 6.0 to obtain a 5 μM final protein concentration. Samples were incubated for 30 min or 2 h, and absorbance spectra between 200 and 600 nm were detected using a double-beam Shimadzu (Kyoto, Japan) UV-1800 UV/Vis spectrophotometer.

### Spectrophotometric HP titration

A fresh 1 mM HP solution was prepared by diluting 10 mM HP stock solution (MedChem Express, Monmouth Junction, NJ, USA) in 100% DMSO. The required amount of HP 1 mM solution (0–10 μL) supplemented with 100% DMSO to a final volume of 10 μL was mixed either with 990 μL of the 50 mM sodium phosphate buffer, pH 6.0 or with the appropriate amounts of the full-length wtBlc protein and 50 mM sodium phosphate buffer, pH 6.0 to obtain a 5 μM final protein concentration. Samples were mixed, and absorbance spectra between 200 and 600 nm were detected using a double-beam Shimadzu UV-1800 UV/Vis spectrophotometer. Sample preparation was performed with all possible precautions to minimize light exposure of the sample and therefore prevent photoinduced changes.

### Tryptophan fluorescence quenching assay

Fresh HP solutions (100, 12.5, 10, and 1.6 μM) were prepared by diluting 10 mM HP stock solution (MedChem Express) in 100% DMSO. The required amount of HP solution (0.5–3 μL) supplemented with 100% DMSO, if needed, to a final volume of 3 μL was mixed with the appropriate amounts of the full-length wtBlc protein and 50 mM sodium phosphate buffer, pH 6.0 to obtain 10 nM final protein concentration. Fluorescence emission spectra were acquired using a Horiba Jobin Yvon Fluoromax-3 fluorometer in the 300–550 nm range with excitation at 290 nm and a step size of 0.25 nm. Sample preparation was performed with all possible precautions to minimize light exposure of the sample and therefore prevent photoinduced changes.

### *K*_d_ calculation

Assuming 1 : 1 protein:ligand binding stoichiometry, the protein–ligand complex concentration in equilibrium ([PL]_eq_) can be expressed as following:

(1) $${\left[\text{PL}\right]}_{\text{eq}}=\frac{\left({\left[L\right]}_{\text{init}}+{\left[P\right]}_{\text{init}}+K_{\text{d}}\right)-\sqrt{{\left({\left[L\right]}_{\text{init}}+{\left[P\right]}_{\text{init}}+K_{d}\right)}^{2}-4{\left[L\right]}_{\text{init}}{\left[P\right]}_{\text{init}}}}{2}$$

where [L]_init_ and [P]_init_ are initial concentrations of a ligand and a protein, and *K*_d_ is the dissociation constant. Then, the total absorption at 411 nm (A_411_) of the protein–ligand solution in equilibrium can be calculated as follows:

(2) $$A_{411}={\left[\text{PL}\right]}_{\text{eq}}\times \varepsilon_{\left[\text{PL}\right]}+\left({\left[\text{L}\right]}_{\text{init}}-{\left[\text{PL}\right]}_{\text{eq}}\right)\times \varepsilon_{\left[\text{L}\right]}$$

where ϵ_[PL]_ and ϵ_[L]_ are extinction coefficients of, correspondingly, the protein–ligand complex and the free ligand at 411 nm at that concentration.

The total fluorescence intensity at 354 nm (I_354_) of the protein–ligand solution in equilibrium can be calculated as follows:

(3) $$I_{354}={\left[\text{PL}\right]}_{\text{eq}}\times I_{\left[\text{PL}\right]}+\left({\left[\text{P}\right]}_{\text{init}}-{\left[\text{PL}\right]}_{\text{eq}}\right)\times I_{\left[\text{P}\right]}$$

where I_[PL]_ and I_[P]_ are fluorescence intensities of, correspondingly, the protein–ligand complex and the free protein at 354 nm when excited at 290 nm.

To determine the apparent *K*_d_, the experimentally obtained data were fitted to the Equation (2) or (3) by the *leastsq* method from the Scipy optimize package. Extinction coefficients of the free ligands were obtained by fitting experimental data of the free ligand absorbance spectra to the polynomial (in the case of the heme titration; Fig. S1A) or linear (in the case of the HP titration; Fig. S1B) function.

### Crystallization, data collection, and structure determination

WtBlc-split (12 mg·mL^−1^ in 50 mM sodium phosphate buffer, pH 6.0) was crystallized at 21 °C in 1.6 M ammonium sulfate, 0.1 M MES, pH 4.5 with protein to precipitant volume ratio of 1 : 1 or in 1.6 M ammonium sulfate, 0.1 M MES, pH 4.5 supplemented with 0.1 M Iron(III) chloride hexahydrate or 5% w/v n-Dodecyl-β-D-maltoside according to the Hampton Research Additive Screen protocol using the sitting drop vapor diffusion technique. Crystals grew within 1 week and were flash frozen in liquid nitrogen using Parabar 10312 oil as cryoprotectant.

Diffraction data were collected at the Life Sciences Collaborative Access Team beamline 21-ID-F at the Advanced Photon Source, Argonne National Laboratory. The diffraction data were processed using the XIA2 software suite [22]. The crystal structures were solved by molecular replacement with MOLREP [23] using the DiB2-split structure as a search model (PDB ID 6UKL). Model building and iterative refinement were performed with COOT [24] and REFMAC [25], respectively. The final statistics of the structure are shown in Table S1. The model has been deposited into the Protein Data Bank (PDB ID 6VRI). Structure figures were prepared using PYMOL (v.2.2.3; Schrödinger, LLC, New York, NY, USA).

### Molecular modeling

#### Model building and protein–ligand docking

Starting coordinates for hematoporphyrin (HP) are based on the common atom coordinates in the crystallographically determined pose of heme in complex with wtBlc-split fit with a maximum common substructure alignment [26]. Split site residues were remodeled with Rosetta comparative modeling (RosettaCM) using a previously solved crystallographic structure of wtBlc (PDB ID 1QWD) to obtain a model of the intact wtBlc. Subsequently, complexes of each ligand with wtBlc were relaxed into the Rosetta Talaris2014 score function with constant starting coordinate constraints.

Following relaxation, we performed local docking-based refinement of wtBlc:HP with RosettaLigand. Small molecule conformers were pregenerated with the BioChemical Library (BCL) cheminformatics toolkit [27], and 2000 rounds of local docking (conformer swap, high-resolution docking, and minimization) were performed starting from the crystallographically determined pose of wtBlc-split: heme.

#### Molecular dynamics (MD) simulations

The best scoring pose of wtBlc:HP refined in RosettaLigand was used to initiate all MD simulations. Amino acid protonation states of wtBlc were assigned by H++ (http://biophysics.cs.vt.edu/H++) [28] at pH 6.0 to match the conditions of the HP titration experiments. HP geometry optimization and electrostatic potential (ESP) calculations were performed in Gaussian [29] at the B3LYP/6–31G* level of theory. Restrained ESP fitting was subsequently performed with AmberTools18 [30] to parameterize HP for classical MD simulations. Full system parameterization was completed using Leap in AmberTools18 [30]. Simulations were performed in Amber18 [30] with the ff14SB force field [31] and the generalized Amber force field 2 (GAFF2) [32]. Structures were solvated in a rectangular box of TIP3P explicit solvent neutralized with Joung–Cheatham monovalent ions [33,34]. wtBlc:HP was buffered on all sides with 12.0 Å solvent. Hydrogen mass repartitioning was performed on solute atoms to allow a simulation timestep of 4.0 fs [35].

Minimization proceeded in three stages: (a) The system was minimized with 500 cycles of steepest descent followed by 1000 steps of conjugate gradient descent (CGD) while protein atoms were restrained with a force constant of 10.0 kcal·mol^−1^·Å^−2^; (b) the system then underwent 500 cycles of steepest descent followed by 1000 steps CGD minimization in buffer restrained with a force constant of 5.0 kcal·mol^−1^·Å^−2^; (c) finally, restraints were removed from the system for 500 steps steepest descent followed by 1000 steps of CGD minimization.

Postminimization, SHAKE [36] and SETTLE [37] (for rigid water molecules) were implemented to constrain covalent bonds to hydrogen atoms. Systems were slowly heated in the canonical (NVT; constant number of particles, temperature, and volume) ensemble to 100 K over 50 ps with a 1 fs timestep. Subsequently, systems were heated in NPT ensemble at 1 bar with isotropic position scaling from 100 to 300K over 500 ps and 1 fs timestep. Equilibration/production simulations were run in the isothermal–isobaric (NPT) ensemble at 300K for 2.0–4.0 μs with a Monte Carlo barostat and integrated every 4 fs. Temperature was controlled using Langevin dynamics with a collision frequency of 1 ps^−1^. Periodic boundary conditions were imposed on the system throughout heating and equilibration/production. Electrostatics were evaluated using the Particle Mesh Ewald method and a distance cutoff of 8.0 Å. Trajectory RMSDs were calculated using CPPTRAJ [38].

### Markov model analysis of molecular dynamics simulations

We constructed a hidden Markov state model (HMM) distinguishing five metastable states of wtBlc:HP using PYEMMA version 2.5.7 [39]. We combined all 12.0 μs of MD simulations and computed pairwise distances between all heavy atoms of HP and all heavy atoms of wtBlc residues within 4.0 Å of any HP atom for each trajectory frame to generate our feature trajectory. Subsequently, we performed time-lagged independent component analysis of the feature trajectory with a lag time of 10 ps. We then constructed an HMM and computed the stationary distribution of each metastable state.

### Binding free energy calculations

The estimated binding free energy between each of the wtBlc:HP states was computed with the molecular mechanics Poisson–Boltzmann surface area (MM-PBSA) and interaction entropy (IE) approaches using the MM-PBSA.py program [40]. From each metastable state identified in the HMM, we randomly resampled 1000 structures to use for wtBlc:HP binding free energy calculations. For the MM-PBSA calculations, the internal and external dielectric constants were set to 4.0 and 80.0, respectively. The nonpolar component of the solvation-free energy was estimated from the solvent accessible surface area with the classical method (INP = 1) using default coefficient and offset values. Atomic radii were taken from the parameter–topology file (RADIOPT = 0). The IE was computed as a *post hoc* analysis of the MM-PBSA gas phase (electrostatic and Van der Waals) contributions to the interaction energy between the protein and ligand, as described by Duan *et al*. [41].

## Results and Discussion

During our previous work on designing a new class of fluorogen-activating proteins named DiBs based on modified lipocalin Blc [19], we excluded protein variants with intensive visible coloring prior to the addition of fluorophores. The wtBlc was one of these excluded variants (Fig. 2A) despite its promising ability to bind some of the tested fluorophores because neither size-exclusion chromatography nor continuous dialysis was able to extinguish or decrease the intensity of the color. We later created a DiB-based split protein reporter by cleaving the protein into two parts [20]. The resultant N- and C-terminal fragments were either (a) genetically fused to leucine zipper peptides and co-expressed (further referred as split-Zip proteins), or (b) co-expressed without additional modifications (further referred as split proteins) (Fig. S2). Based on the proximity of the cleavage site to the binding pocket, we hypothesized that split wtBlc could show reduced binding to the unknown colored compound. Correspondingly, we created and examined two similar constructs generated from the wtBlc: wtBlc-split-Zip and wtBlc-split. Counter to our hypothesis, both wtBlcsplit-Zip and wtBlc-split retained their intense brownish color when expressed in LB medium.

**Fig. 1.**
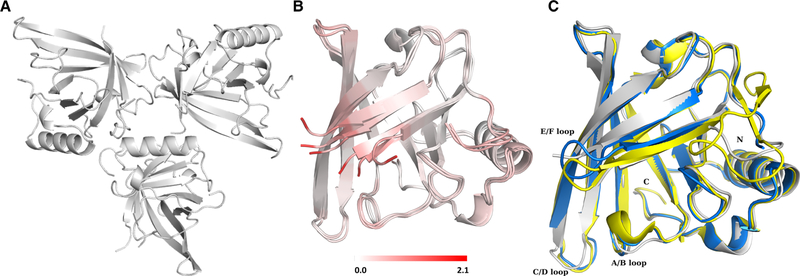
Overall wtBlc-split structure characterization. Cartoon representation of the (A) wtBlc-split crystal asymmetric unit, (B) overlaid three wtBlc-split nonsymmetric monomers, and (C) a wtBlc-split monomer (grey) aligned with previously determined full-length Blc structures (3MBT, blue; 2ACO, chain A, yellow). (B) Residues of the overlaid monomers are color-coded according to their distance to the mean position of three equivalent residues. Only residues that are present in all three nonsymmetric monomers are shown. Pairwise Cα rmsd (150 atoms) equal to 0.6, 1.2, and 1.2 Å. (C) Structure of the third full-length Blc structure, 1QWD, almost perfectly overlays with 2ACO (rmsd = 0.1 Å) and therefore is not shown in order to reduce the complexity of the figure

Attempts to obtain crystals of the colored full-length wtBlc protein were unsuccessful. However, we were able to determine the structure of wtBlc-split protein at 1.94 Å resolution. The wtBlc-split asymmetric unit cell contained three ‘split’ molecules (Fig. 1A) that differ primarily in the area of the two β-strands adjacent to the split point (Fig. 1B). Two fragments of the split protein together fully reconstitute the typical lipocalin fold. Eight antiparallel β-strands (designated A–H) form a barrel and are accompanied by a C-terminal α-helix. Alignment of a wtBlc-split ‘monomer’ with previously determined Blc structures (PDB IDs 1QWD, 2ACO, and 3MBT) reveals no significant structural perturbation. The Cα root mean-squared deviation (rmsd) between this structure and those previously reported range from 0.98 Å (3MBT) to 1.02 Å (2ACO) over 147 shared residues. Structural deviations are primarily located in the area adjacent to the split protein cleavage point (former E/F loop). Other deviations can be observed in two other loops (A/B and C/D loops) as well as at the N-terminus (Fig. 1C). These areas colocalize with the previously identified regions where the full-length Blc structures differ from each other [16] and most likely reflect the overall flexibility of the long loops.

**Fig. 2.**
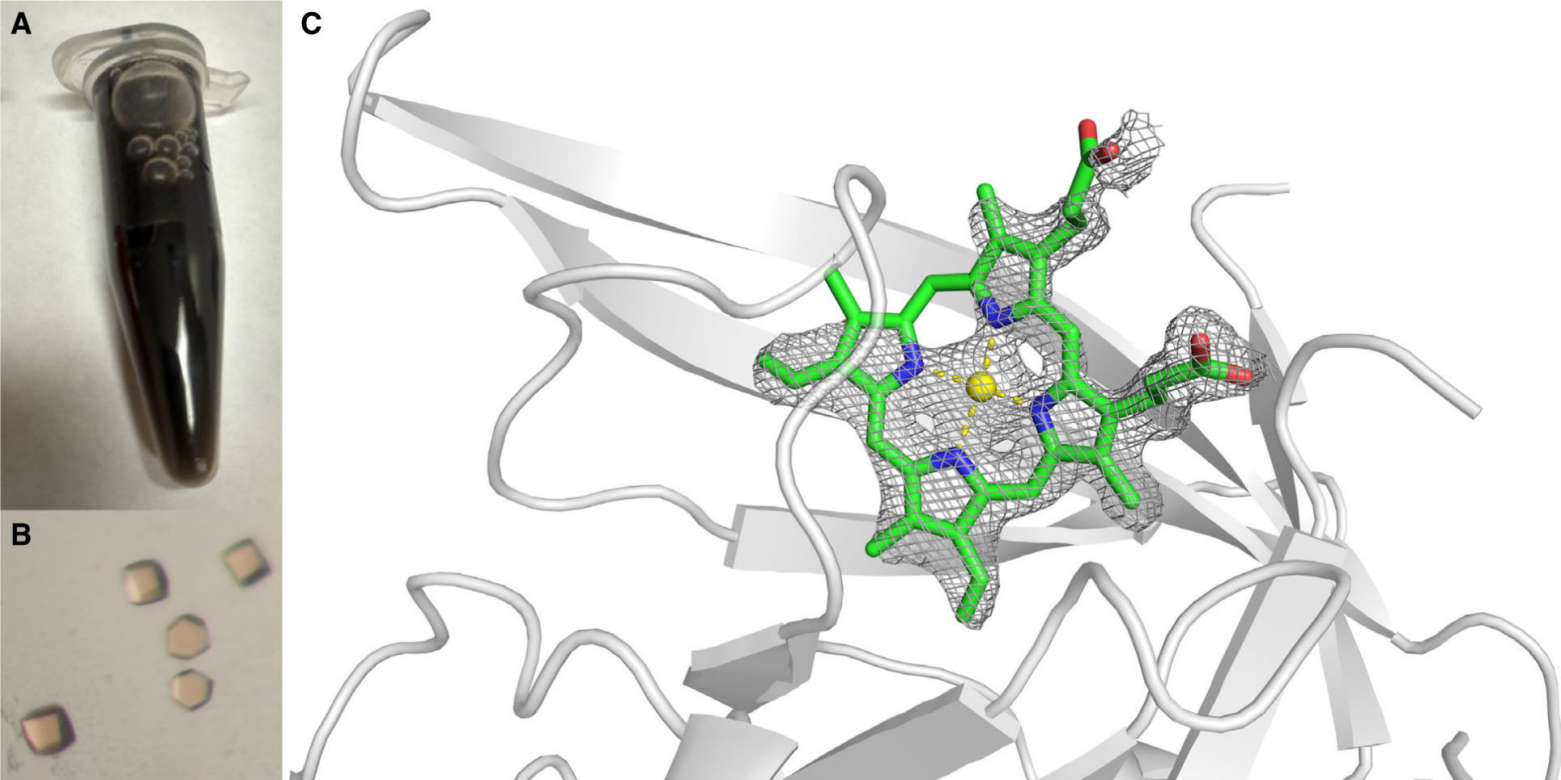
Evidence for the presence of heme in the Blc protein. (A) The concentrated (~ 50 mg·mL^−1^) solution of the purified wtBlc protein. (B) Colored ‘apo’ wtBlc-split crystals. (C) Heme-like density in the wtBlc-split crystal. The 2*F*_o_-*F*_c_ electron density map from the binding pocket region of one of the wtBlc-split protein molecules after omit refinement is shown as grey mesh. The protein molecule is shown as gray cartoon. The putative heme ligand is shown as sticks.

Consistent with the colored protein solution, the wtBlc-split crystals also had notable color (Fig. 2B). During structure refinement, we discovered a well-defined positive difference density map area in the binding pocket of one of the three ‘split’ molecules in the asymmetric unit. Based on the shape of the density as well as previous observations including color of the protein, absorbance, and fluorescence spectra, we suggested the presence of the heme B molecule in the structure (Fig. 2C). To test our hypothesis that heme B is present in the wtBlc-split structure, we performed a number of experiments.

**Fig. 3.**
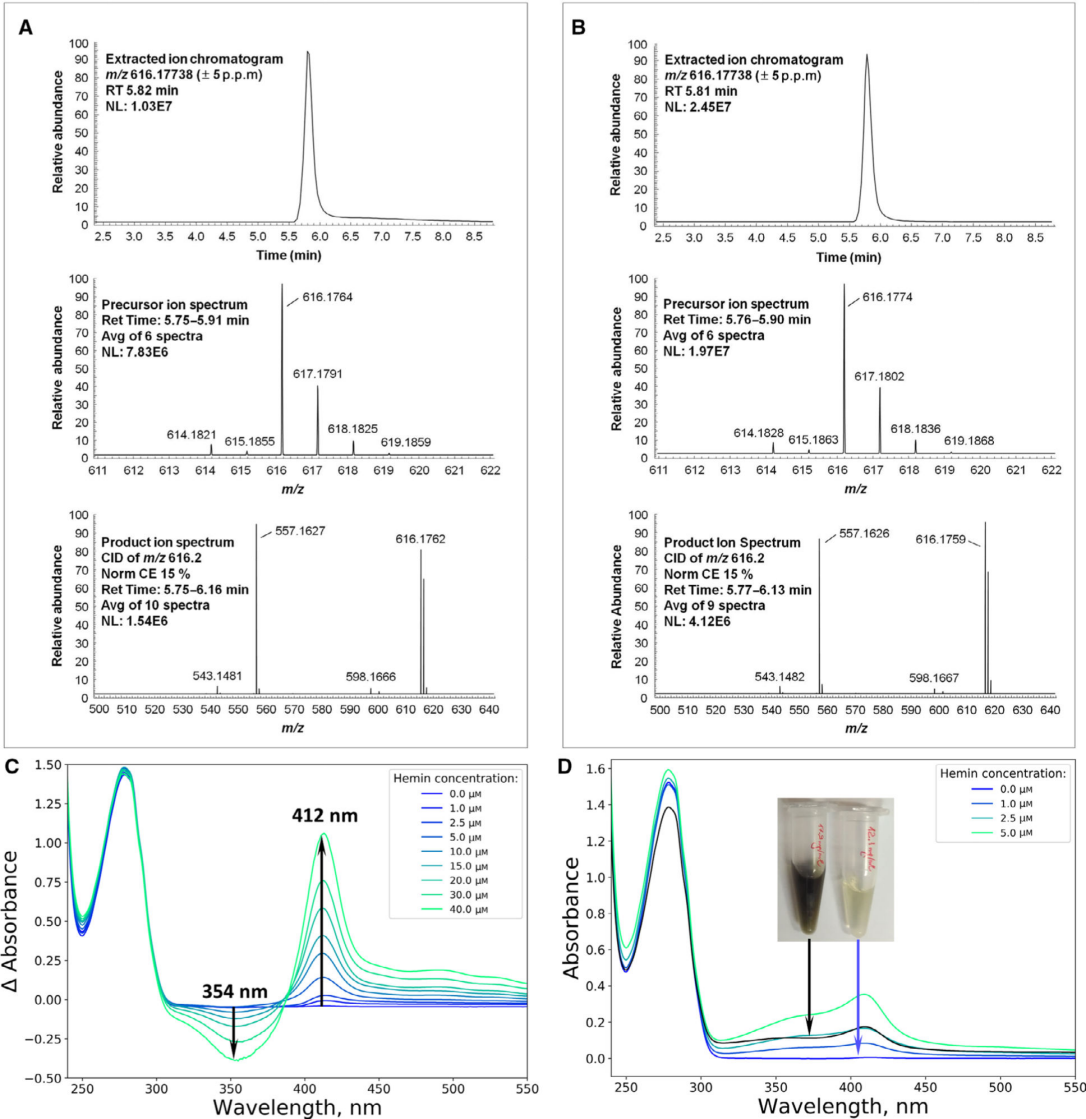
Confirmation of the presence of heme in the Blc protein. (A-B) Detection of ferric heme by HPLC-high resolution MS and MS/MS in (A) 1 mM hemin chloride (solution in 100% DMSO) standard diluted in H_2_O/MeOH (1 : 1) to a final concentration of 10 μM, 100 pmol hemin on column. (B) 0.5 mM heterologously expressed wtBlc-split (solution in 50 mM sodium phosphate buffer, pH 6.0) diluted in H_2_O/MeOH (1 : 1) to a final concentration of 5 μM, 50 pmol protein on column. (C) Differential absorbance spectra of heme bound to M9-expressed wtBlc protein. Increasing aliquots of 1 mM hemin solution in DMSO were added to 40 μM protein solution and measured using the mixture of the same amount of the hemin solution with the buffer as a reference read. (D) Absorbance spectra of wtBlc protein expressed in minimal medium and supplemented with heme in comparison with the spectrum of wtBlc protein expressed in LB (black solid line). Inset: difference in the color of the solutions of the purified wtBlc protein samples expressed in LB (left) and minimal medium (right).

First, we compared the wtBlc-split protein sample used for growing crystals with the commercially available hemin standard (≥ 97.0% HPLC; Sigma-Aldrich) using HPLC-high-resolution MS and MS/MS. LC-MS/MS analysis of the wtBlc-split protein sample showed the presence of a small molecule with a [M + H]^+^ *m/z* = 616.1774, having the same chromatographic retention time, precursor ion exact mass, precursor ion isotopologue pattern, and CID product ion spectrum of the standard ([M + H]^+^ *m/z* = 616.1764; Fig. 3A,B).

Second, we used absorption spectroscopy. During our work, we discovered that the recombinant protein expression in minimal medium (M9 salts) results in much less-colored protein samples (Fig. 3D, inset). We titrated hemin into the full-length wtBlc recombinant protein expressed in minimal medium and compared these spectra with absorption of free hemin in the buffer. The resulted differential spectra demonstrated gradually decreasing absorption at ~ 350 nm and increasing absorption at 412 nm (Fig. 3C). The latter absorption peak is known as the Soret peak and is characteristic for heme-binding proteins [42]. Notably, the spectrum of the protein expressed in minimal medium supplemented with hemin matches almost perfectly the absorbance of the LB-expressed wtBlc protein (Fig. 3D). However, this comparison also suggests that the LB-expressed wtBlc samples are not saturated with heme. This may explain the presence of the heme molecule only in one out of three copies of the split protein in the asymmetric unit in the obtained crystal structure.

**Fig. 4.**
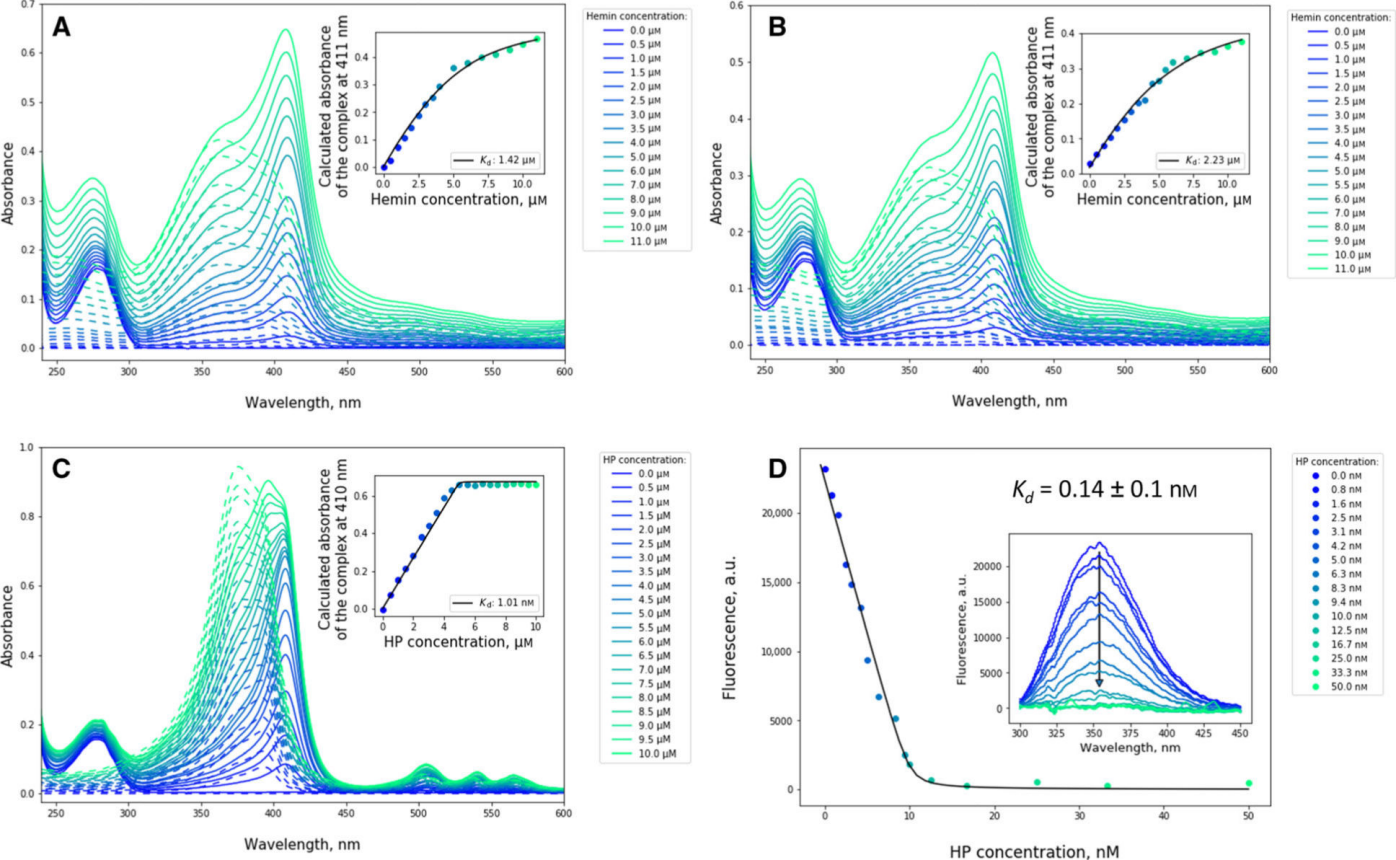
wtBlc binding to porphyrins. Representative examples of the spectrophotometric heme titration of wtBlc (A) and wtBlc-split (B). Solid lines show absorbance spectra of 5 μM protein solution (expressed in minimal medium) supplemented with increased concentrations (0.5–11 μM) of heme. Dashed lines show absorbance spectra of increased concentrations (0.5–11 μM) of heme in the buffer. Inset: the calculated absorbance of the formed protein:heme complex at 411 nm and the fitting curve (black line). (C) A representative example of the spectrophotometric HP titration of wtBlc. Solid lines show absorbance spectra of 5 μM full-length minimal medium expressed wtBlc protein solution supplemented with increased concentrations (0.5–10 μM) of HP. Dashed lines show absorbance spectra of increased concentrations (0.5–10 μM) of HP in the buffer. Inset: the calculated absorbance of the formed wtBlc:HP complex at 410 nm and the fitting curve (black line). (D) A representative example of the fluorometric HP titration of wtBlc. Data points show tryptophan fluorescence intensity at 354 nm of 10 nM full-length minimal medium expressed wtBlc protein solution supplemented with increased concentrations (0.8–50 nM) of HP. The fit of the emission intensity to Equation (3) (black line) yielded a *K*_d_ of 0.14 ± 0.1 nM. Inset: tryptophan fluorescence spectra of 10 nM full-length minimal medium expressed wtBlc protein solution supplemented with increased concentrations (0.8–50 nM) of HP. The arrow shows the direction of the changes as the concentration of HP increases.

After confirming our initial hypothesis of the presence of heme in the binding pocket of the wtBlc-split protein, we proceeded with a more detailed characterization of the discovered interaction. The heme molecule occupied the same hydrophobic cavity of the lipocalin as it was previously showed for vaccenic acid [14] (Fig. S3). We further assessed the hemin-binding affinity to the wtBlc. For this, we used the full-length wtBlc protein expressed in minimal medium. This sample failed to have any detectable absorption at 411 nm and according to the LC-MS contained negligible amounts of copurified heme. We measured absorbance spectra of the protein in the presence of different hemin concentrations (Fig. 4A). Assuming 1 : 1 protein : ligand binding stoichiometry based on the obtained crystal structure, spectrophotometric titration yielded a *K*_d_ of 1.4 ± 0.3 μM. This value is comparable to those determined for full-length wtBlc binding to fatty acids [14] or some other heme-binding proteins like lipocalin α_1_-microglobulin [43], bacterial chlorite dismutase-like protein HemQ [44], or some all-helical hemoproteins like horseradish peroxidase [45]. The affinity for heme is lower than that of classical heme-binding proteins like apohemoglobins and apomyoglobins (*K*_d_ on the order of 10^−14^ M) [46], some other β-barrel proteins like nitrobindins (*K*_d_ on the order of 10^−12^ M) [47], or hemophores (*K*_d_ on the order of 10^−10^ M) [48].

To further ensure that splitting of wtBlc did not fundamentally affect its ability to bind to heme and, therefore, that we can extrapolate our wtBlc-split:heme complex-based findings onto the full-length protein, we also performed the same spectrophotometric heme titration of the wtBlc-split protein. This titration yielded a similar dissociation constant of 2.0 ± 0.3 μM (Fig. 4B).

**Fig. 5.**
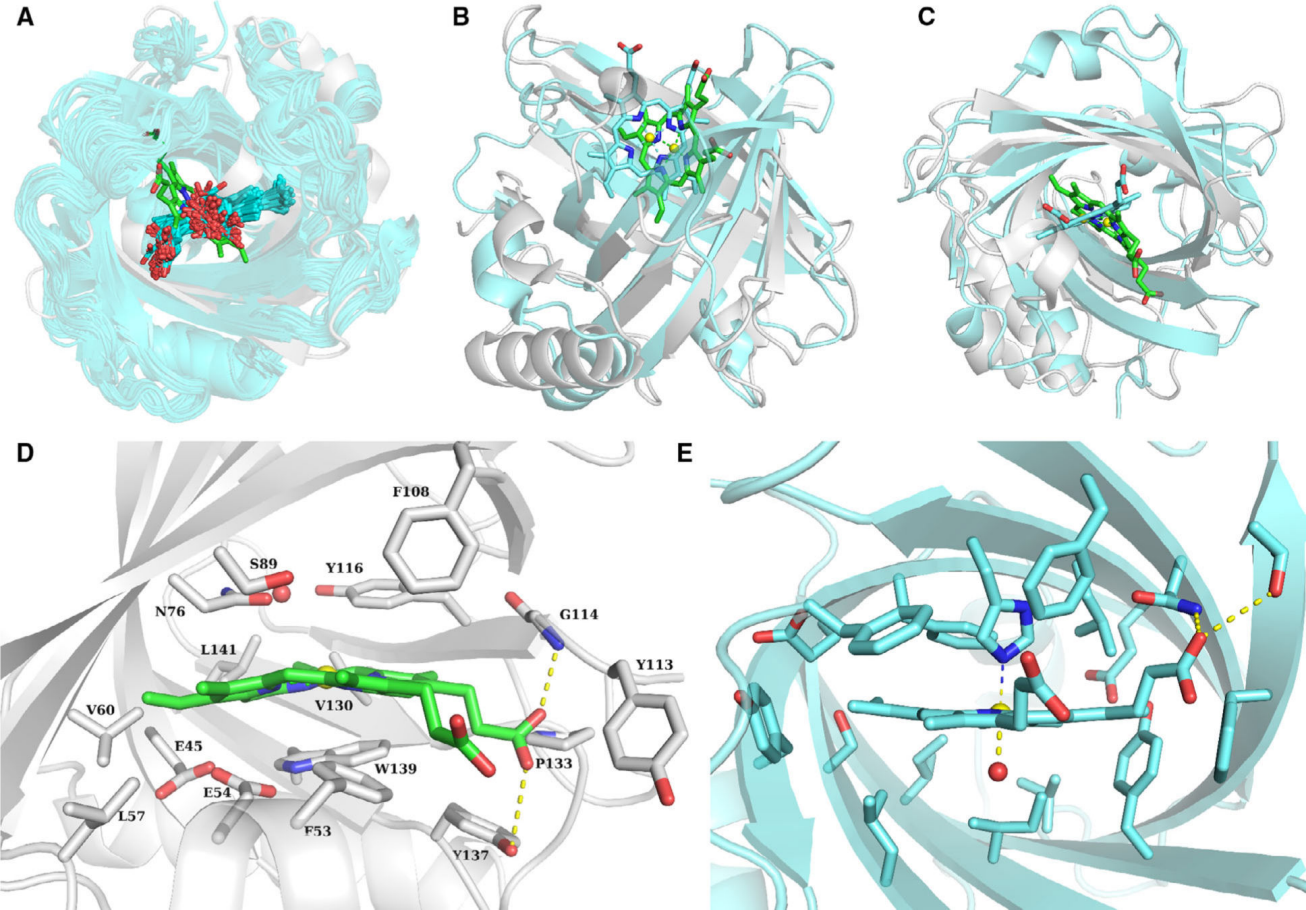
Comparison of heme binding and coordination by wtBlc-split with other lipocalins cocrystallized with heme. (A) Overlay of 67 lipocalin structures cocrystallized with heme found in the PDB (proteins are shown as cyan cartoon, and heme molecules are shown as cyan sticks) aligned with wtBlc-split structure (protein is shown as grey cartoon, and heme is shown as green sticks). The position of heme in the binding pocket of wtBlc-split (grey cartoon, heme is shown as green sticks) and nitrophorin 7 (PDB ID 4XMC, protein is shown as cyan cartoon, and heme is shown as cyan sticks): (B) side view and (C) view from the top. (D) Heme coordination in the binding pocket of the wtBlc-split protein. The protein molecules are shown as grey cartoon. Amino acid side chains within 4 Å distance from the ligand are shown as sticks. Heme is shown as green sticks. Hydrogen bonds are shown as yellow dashed lines. (E). Classical heme coordination in the binding pocket of nitrophorin 7 (PDB ID 4XMC). Hydrogen bonds are shown as yellow dashed lines.

We then compared the heme binding and coordination by wtBlc-split with other known structures. We downloaded all available structures of lipocalins cocrystalized with heme from the PDB. All 67 of these belong to the nitrophorins family. While all proteins superimposed relatively well (~ 4 Å rmsd over backbone atoms), position of heme in the binding pocket in wtBlc-split is strikingly different from all other analyzed heme-binding lipocalins (Fig. 5A). Taking into account the overall similarity of all found nitrophorins, we continued comparison of the heme-binding modes of wtBlc-split and nitrophorins using one structure, nitrophorin 7 from *Rhodnius prolixus* (PDB ID 4XMC) [49], which has the lowest overall rmsd to wtBlc-split. In both structures, heme occupies the main cavity of the lipocalin fold and is situated similarly deep in the binding pocket (Fig. 5B). However, the plane of the heme molecule in the wtBlc-split binding pocket is rotated almost 120 degrees relative to its position in nitrophorin 7 (Fig. 5C).

In the binding pocket formed by chains E and F of the structure, there are 19 amino acid residues containing atoms within a 4.0 Å distance from the ligand (Glu45, Phe53, Glu54, Leu57, Val60, Asn76, Lys77, Gly78, Ser89, Phe108, Tyr113, Gly114, Gly115, Tyr116, Val130, Pro133, Tyr137, Trp139, and Leu141). Phe53 and Trp139 form π-stacking interactions with the porphyrin rings IV and II. The side-chain hydroxyl of Tyr137 and backbone amide of Gly113 stabilize one of the propionate groups of heme by hydrogen bonds. All other listed amino acids contribute to the hydrophobicity of the binding pocket (Fig. 5D). Heme also interacts with two water molecules—one at the solvent front and one buried near Y116 (Fig. S4). There are two additional amino acid residues from the adjacent ‘split’ molecule in the asymmetric unit (Ser148 and Glu150 from chain D) that contribute interactions with heme at the solvent front (Fig. S4). These latter amino acid contacts may be sterically impaired in the full-length wtBlc with the intact E/F loop. Moreover, according to the Protein Interfaces, Surfaces and Assemblies (PISA) server [50] evaluation, the interface between ‘split’ molecules in the trimer is unlikely to be stable in solution. This suggests that the observed interactions of the heme propionate group with the amino acids from chain D are most likely the result of the crystal packing. We speculate that in solution the propionate group is exposed to water.

While the presence of heme propionate anchors as well as the described above heme ‘face’ and heme edge interactions are standard interactions that were previously reported in other heme-binding proteins [51], the classical heme iron atom coordination by protein amino acid(s) (proximal ligand interaction) is missing, making the observed heme-binding mode unusual (Fig. 5E). We considered that this heme-binding mode may be a crystallographic artifact. To investigate the stability of the observed binding pose, we employed MD simulations. We performed three independent 2.0 μs simulations of wtBlc-split:heme and the modeled full-length wtBlc:heme complexes. In all six simulations, we saw no substantial deviation from the crystallographic pose (Fig. S5A). Superimposition of the binding pockets from the final frame of both the wtBlc-split:heme (Fig. S5B) and wtBlc:heme (Fig. S5C) trajectories shows good overlap of heme with the crystallographically determined pose. These results support our experimental structural data and suggest that the observed wtBlc-split:heme interaction in the crystal structure is not a crystallographic artifact.

However, wtBlc may not have evolved specificity toward heme binding and may bind other porphyrins. To test this hypothesis, we evaluated the ability of wtBlc to interact with other chemically similar porphyrins: protoporphyrin IX, and non-natural porphyrins HP and verteporfin (Fig. S6).

Of the three tested compounds, only HP showed significant perturbations in absorbance spectra upon adding the protein. To characterize the HP binding, we repeated an absorbance titration using a constant concentration of the full-length wtBlc protein expressed in minimal medium supplemented with different HP concentrations (Fig. 4C) analogous to the previously performed hemin titration. Based on similar size and overall shape of the heme and HP molecules, we hypothesized the same 1 : 1 protein : ligand ratio of the wtBlc : HP interaction. Data analysis suggested a *K*_d_ in the low nM range, but the sensitivity limitations of the method did not allow for accurate *K*_d_ determination. For more precise *K*_d_ determination, we exploited the tryptophan fluorescence quenching assay that was previously used for potential Blc ligand screening [14]. The analysis yielded a *K*_d_ of 0.14 ± 0.1 nM (Fig. 4D).

HP differs from heme in two key ways: (a) HP contains no coordinated iron, and (b) the vinyl groups in heme are hydrated to alcohols in HP. Nevertheless, they are structurally similar porphyrins, and it is not immediately clear why HP has a substantially higher binding affinity to wtBlc than heme. To further investigate this observation, we performed *in silico* analysis. First, we used Rosetta to construct a model of the wtBlc:HP complex such that HP was oriented to its common substructure with heme. We then performed three independent 4.0 μs MD simulations of wtBlc:HP to evaluate stability of HP in the binding pocket.

To our surprise, HP consistently departed from its initial heme-like binding mode to rotate ~ 90°, orienting both of the propionate groups toward the solvent front. To identify the most frequently occupied HP-binding modes, we generated HMM from the simulations of the wtBlc:HP complex. Our HMM identified five HP-binding modes: one heme-like binding mode (state 1), three variants of the 90-degree rotated binding mode (states 0, 3, and 4), and one intermediate binding mode (state 2) (Fig. S7). The transition to states 0, 3, and 4 occurred between 0.8 and 2.0 μs (Fig. S8). Based on the relative free energy profiles and stationary distributions of each state in the HMM (Fig. S7A) as well as MM-PBSA-IE binding free energy calculations of structures resampled from each HMM state (Table S2), we chose wtBlc:HP state 4 model for detailed comparison with wtBlc-split:heme crystal structure.

**Fig. 6.**
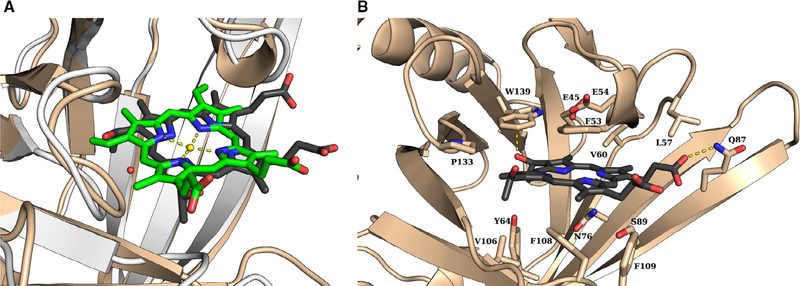
Comparison of the converged pose of wtBlc:HP MD simulations with the crystal structure of wtBlc-split:heme. (A) Overlay of a randomly resampled wtBlc:HP pose from HMM state 4 (dark gray sticks and wheat cartoon) and the co-crystalized wtBlc-split:heme (green sticks and white cartoon; ferric iron is depicted as a yellow sphere with yellow dashed lines indicating coordination to porphyrin nitrogen atoms; a structural water molecule in the binding pocket is depicted as a red sphere). (B) Interactions between HP and wtBlc in HMM state 4. Yellow dashed lines indicate hydrogen bonds.

The porphyrin rings of heme and HP overlap, which results in involvement of generally the same set of amino acids into HP ‘face’ and HP edge interactions (Fig. 6) as in the wtBlc-split:heme complex (Fig. 5D). Interestingly, one of the HP alcohols overlaps with the heme vinyl situated next to the buried structural water molecule (Fig. 6A), potentially reducing occupancy of water in this site or providing the water molecule with additional hydrogen bonding interactions. The other HP alcohol is oriented in the same direction as one of the heme propionate groups near the ‘split’ site. Both HP propionate groups are directed outward at the solvent front. These observations suggest a possible structural explanation for the observed higher binding affinity of wtBlc to HP relative to heme: The wtBlc: HP complex may benefit from a reduced desolvation penalty.

HP, as well as other porphyrins and their derivatives, is well-known for its photosensitizing properties [52]. While performing titration experiments, we discovered that the presence of the wtBlc protein in solution makes HP incredibly sensitive to light. When the sample was kept in darkness, no changes in the spectra were observed during a 1-h period. However, visible color change of the solution and dramatic absorbance spectral changes happened after the sample was outside of the spectrophotometer chamber for as little as one minute (Fig. S9). Neither of these changes were seen in the absence of the protein in the similar time scale. Further experiments in a nitrogen glove box showed that the observed color change depends on the presence of oxygen. We speculate that wtBlc accelerates the degradation of HP.

## Conclusions and Future directions

Using LC-MS/MS and absorption spectroscopy, we confirmed copurification of the heterologously expressed wtBlc and wtBlc-split proteins with heme. This allowed us to assign the observed ‘heme-like’ density in the obtained wtBlc-split crystal structure as a heme molecule and to investigate wtBlc:heme interaction in more detail.

Based on the spectrophotometric hemin titration experiments and crystal structure analysis, we assessed the wtBlc hemin-binding affinity to be in the low μM range with 1 : 1 ligand-to-protein stoichiometry. While most of the observed protein:heme interactions are similar to those found in other heme-binding proteins, wtBlc-split does not coordinate the iron of the heme molecule. This observation prompted us to test whether wtBlc binds any other heme-like molecules. Interestingly, while the iron-free protoporphyrin IX molecule seems not to interact with the protein according to our absorption spectroscopy experiments, additional hydration of the two vinyl groups as seen in HP not only rescues the binding, but also improves the affinity by approximately three orders of magnitude.

Blc is a membrane-associated protein anchored to the outer membrane by a covalently bound N-terminal cysteine lipid, and it faces the periplasmic space [12,13]. Based on the obtained data, we speculate that lipocalin Blc might participate in the trans-periplasmic trafficking of heme molecules. While sequestering heme from highly competitive extracellular environments requires tight heme binding, further transportation inside a protected space might be performed by a protein with much lower affinity [53]. However, the fact that the wtBlc-split:heme complex has been purified from the unnatural cytoplasmic environment together with the observed high affinity for HP leaves the possibility that the main ligand of the Blc is yet another metal-containing or metal-free tetrapyrrole. There currently exists no evidence that the protein predominantly binds to only one ligand; therefore, Blc may target a broad family of tetrapyrroles, including heme.

The observed light sensitivity of the wtBlc:HP complex was out of scope of the current research and therefore was not closely investigated. However, further study might result in creation of new oxygen sensing indicators, genetically encoded light-induced sources of reactive oxygen species, or tags for correlative light and electron microscopy.

## Supplementary Material

Supp **Table S1.** Data Collection and Refinement Statistics
**Table S2.** Binding free energy estimates of wtBlc:HP in different HMM metastable states.
**Fig. S1.** Absorbance of solutions with different concentrations of free hemin at 411 nm (A) or free HP at 410 nm (B), and fitted curves (black dashed lines).
**Fig. S2.** Schematic representation of the design of the leucine zippers-containing (“split-Zip”) and leucine zippers-free (“split”) DiB-split proteins constructs.
**Fig. S3.** Comparison of different proposed wtBlc ligands’ binding sites.
**Fig. S4.** Heme coordination in the binding pocket of the wtBlc-split protein, side view.
**Fig. S5.** Time-dependent rmsd of heme to its crystallographically determined pose.
**Fig. S6.** Structures of (A) Heme B, (B) protoporphyrin IX, (C) hematoporphyrin, and (D) verteporfin
**Fig. S7.** Metastable states of wtBlc:HP distinguished by a hidden Markov state model (HMM).
**Fig. S8.** HP transitions from the initial heme-like binding mode to an alternative stable pose.
**Fig. S9.** Light-induced changes of the wtBlc:HP solution.

## Abbreviations

CGD: conjugate gradient descent
ESP: electrostatic potential
HMM: hidden Markov state model
HP: hematoporphyrin
MD: molecular dynamics
NO: nitric oxide
PDB: Protein Data Bank
wtBlc: wild-type Blc.
