# Supplementary material for: Lipocalin Blc is a potential heme-binding protein
Source: FEBS Lett. Author manuscript; Available in PMC 2022 Jan 1. (PMC8177097; doi:10.1002/1873-3468.14001)
Supplement: Supp — Table S1. Data Collection and Refinement Statistics Table S2. Binding free energy estimates of wtBlc:HP in different HMM metastable states. Fig. S1. Absorbance of solutions with different concentrations of free hemin at 411 nm (A) or free HP at 410 nm (B), and fitted curves (black dashed lines). Fig. S2. Schematic representation of the design of the leucine zippers-containing (“split-Zip”) and leucine zippers-free (“split”) DiB-split proteins constructs. Fig. S3. Comparison of different proposed wtBlc ligands’ binding sites. Fig. S4. Heme coordination in the binding pocket of the wtBlc-split protein, side view. Fig. S5. Time-dependent rmsd of heme to its crystallographically determined pose. Fig. S6. Structures of (A) Heme B, (B) protoporphyrin IX, (C) hematoporphyrin, and (D) verteporfin Fig. S7. Metastable states of wtBlc:HP distinguished by a hidden Markov state model (HMM). Fig. S8. HP transitions from the initial heme-like binding mode to an alternative stable pose. Fig. S9. Light-induced changes of the wtBlc:HP solution. [file NIHMS1697251-supplement-Supp.pdf]

Supporting information

Lipocalin Blc is a heme binding protein?

Nina G. Bozhanova[1], M. Wade Calcutt[2], William N. Beavers[3], Benjamin P. Brown[1], Eric P. Skaar[3], Jens Meiler[1,4]

[1] *Department of Chemistry, Center for Structural Biology, Vanderbilt University, Nashville, TN, USA*

[2] *Mass Spectrometry Research Center and Department of Biochemistry, Vanderbilt University School of Medicine, Nashville, TN, USA*

[3] *Department of Pathology, Microbiology, and Immunology, Vanderbilt University Medical Center, Nashville, TN, USA*

[4] *Institute for Drug Discovery, Medical School, Leipzig University, Leipzig, SAC 04103, Germany*

Table S1. Data Collection and Refinement Statistics

	**wtBlc-split (PDB ID 6VRI)**
Wavelength (Å)	0.97872
Space group	$P3_221$
Unit cell dimensions	a = b = 68.416,<br>c = 217.748,<br>$\alpha = \beta = 90°$,<br>$\gamma = 120°$
Resolution range (Å)	59.25 - 1.94 (1.97 - 1.94)
Total no. of reflections	403,082 (16,963)
Unique reflections	44,856 (1,915)
Completeness (%)	99.5 (86.2)
Multiplicity	9.0 (8.9)
Mean I / σ(I)	8.1 (2.1)
$CC_{1/2}$	0.992 (0.778)
**Refinement Statistics**	
Rwork/Rfree(%)	21.21/24.9
Average B factor (Å^2)	35.18
Ligand (heme) average B factor (Å^2)	36.54
Total no. of atoms	3,887
Protein atoms	3,707
Ligand (heme) atoms	43
Water molecules	72
Sulphate ions (buffer)	65
Protein residues	457
Bond angles (°)	1.97
Bond length (Å)	0.016
Ramachandran: favored/allowed (%)	98.88/1.12
Clashscore	3.10
Numbers in parentheses are for the highest-resolution shell.	

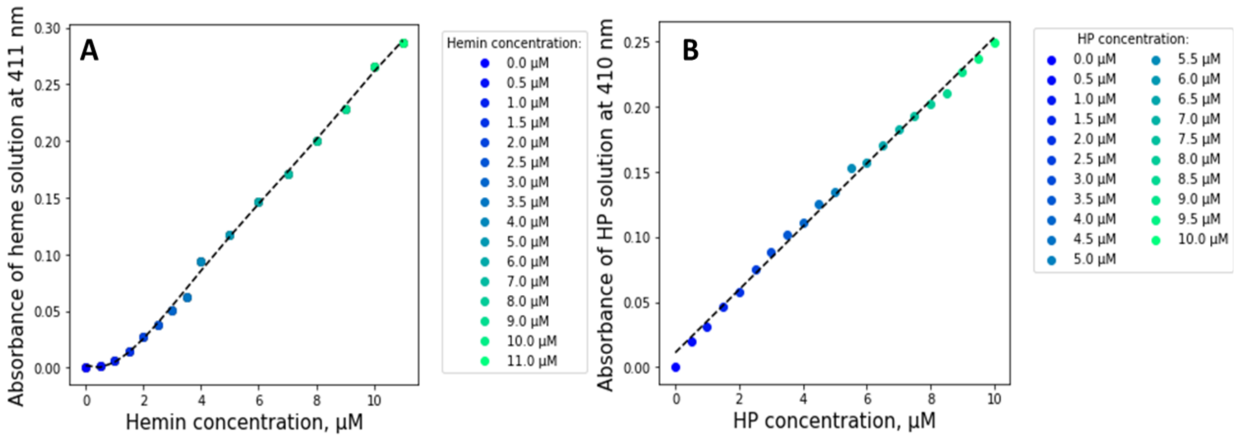

Figure S1. Absorbance of solutions with different concentrations of free hemin at 411 nm (A) or free HP at 410 nm (B), and fitted curves (black dashed lines).

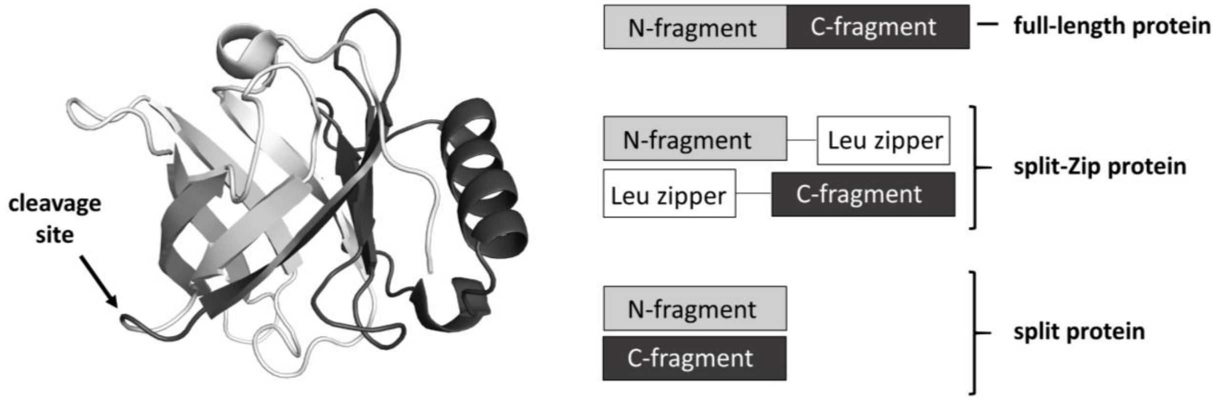

Figure S2. Schematic representation of the design of the leucine zippers-containing ("split-Zip") and leucine zippers-free ("split") DiB-split proteins constructs.

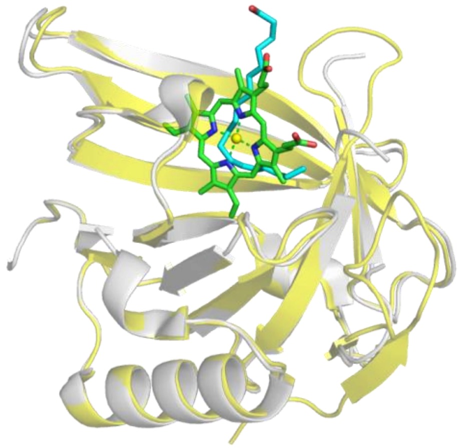

Figure S3. Comparison of different proposed wtBlc ligands' binding sites. The heme molecule (green sticks) occupies the same hydrophobic cavity of wtBlc-split (grey cartoon) as vaccenic acid (cyan sticks) in full-length wtBlc (yellow cartoon, PDB ID 2ACO).

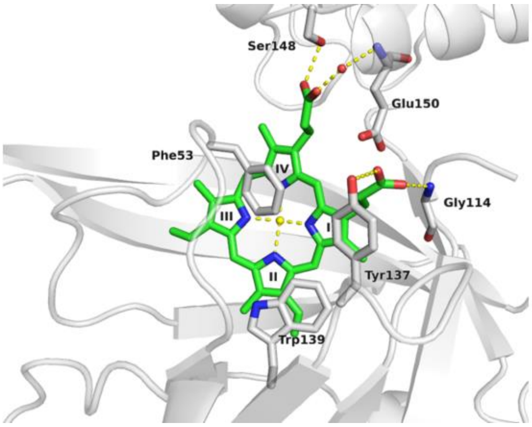

Figure S4. Heme coordination in the binding pocket of the wtBlc-split protein, side view. The protein molecules are shown as grey cartoon. The heme ligand and the sidechains of the most important residues for heme coordination (Phe53, Trp139, Tyr137, Gly113, Ser148, and Glu150) are shown as sticks. Hydrogen bonds are shown as yellow dashed lines.

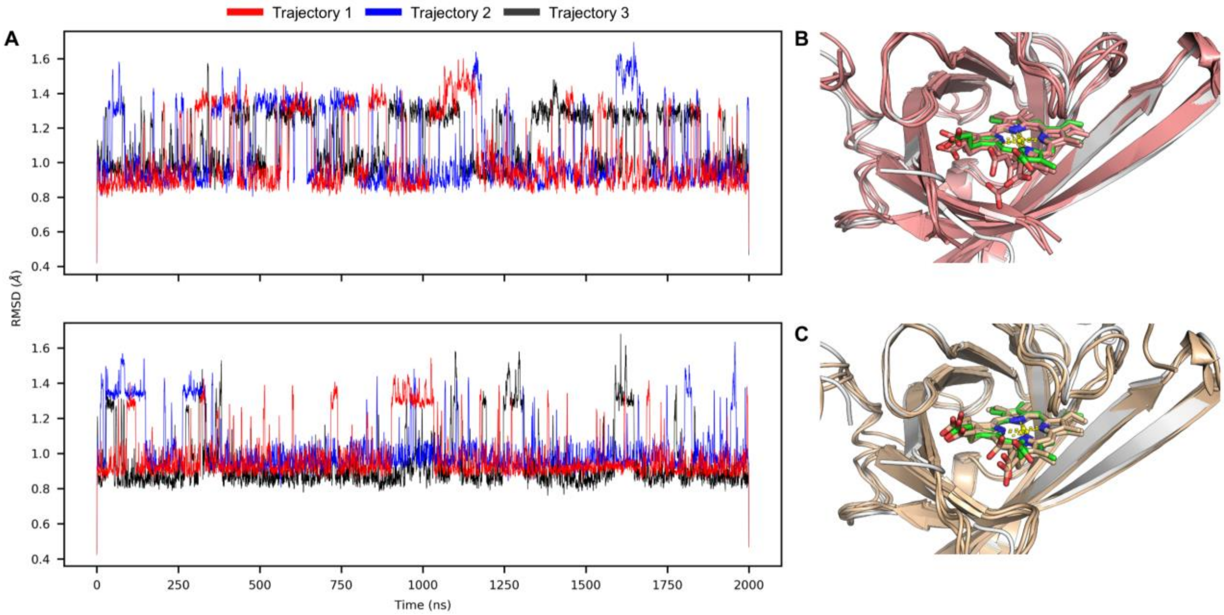

Figure S5. Time-dependent rmsd of heme to its crystallographically determined pose. (A) Rmsd vs. time plots for six independent 2.0 µs MD simulations starting from either wtBlc-split:heme (top) or wtBlc:heme (bottom). (B) Overlay of wtBlc-split:heme after three independent 2.0 µs MD simulations with the co-crystal structure of wtBlc-split:heme. (C) Overlay of wtBlc:heme after three independent 2.0 µs MD simulations with the co-crystal structure of wtBlc-split:heme. All proteins depicted in cartoon format. Crystallographic structure of wtBlc-split protein is colored white. Post-MD simulation structure of wtBlc-split and wtBlc are colored salmon and wheat, respectively. The crystallographic structure of heme is represented in green stick format. Modeled heme is colored either salmon or wheat corresponding the protein structure. Ferric iron is depicted as a yellow sphere with yellow dashed lines indicating coordination to porphyrin ring nitrogen atoms.

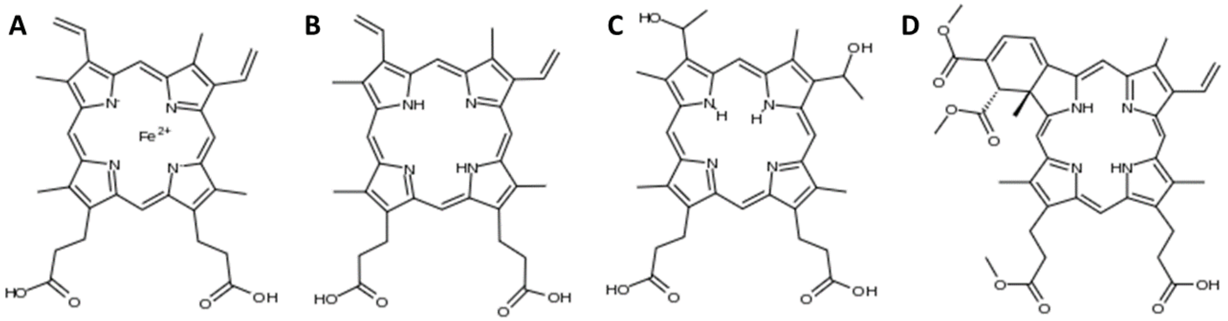

Figure S6. Structures of (A) Heme B, (B) protoporphyrin IX, (C) hematoporphyrin, and (D) verteporfin.

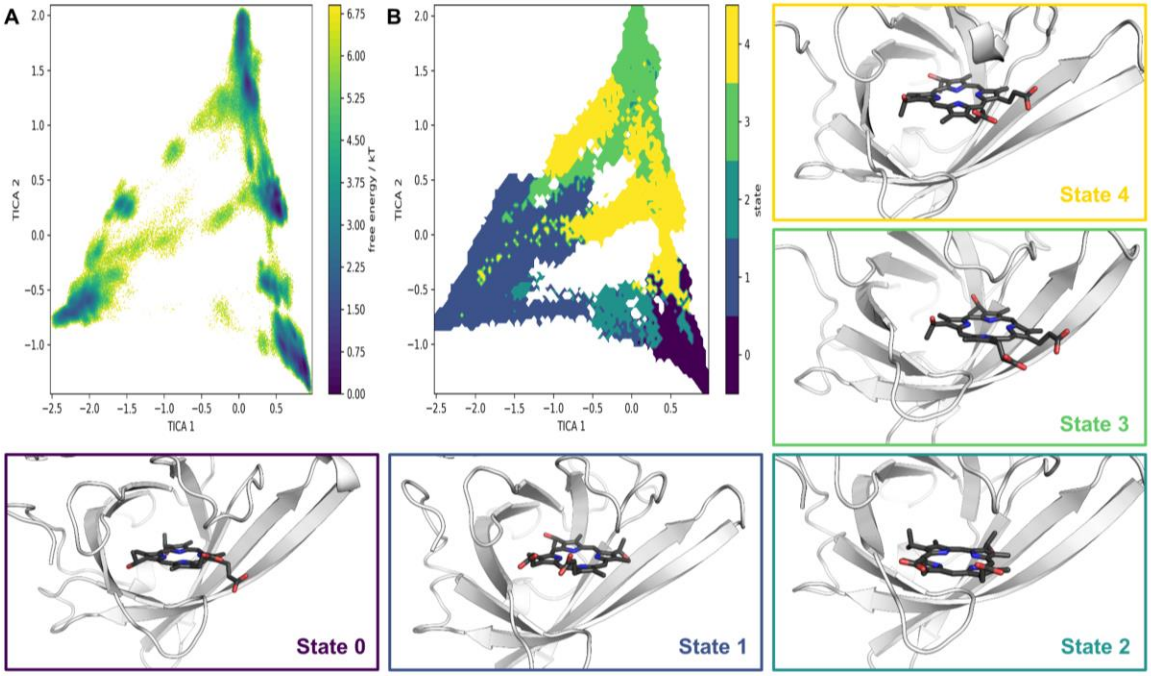

Figure S7. Metastable states of wtBlc:HP distinguished by a hidden Markov state model (HMM). (A) Conformational free energy map of wtBlc:HP projected onto the first two time-lagged independent components. (B) State map of wtBlc:HP projected onto the first two time-lagged independent components. A random snapshot of each state (one heme-like binding mode (state 1; stationary distribution 0.16), three variants of the 90 degree rotated binding mode (states 0, 3, and 4; stationary distributions 0.32, 0.22, and 0.28, respectively), and one intermediate binding mode (state 2; stationary distribution 0.02)) is depicted outlined in the color corresponding to the state map. HMM was built from 3 independent 4.0 μs MD simulations of wtBlc:HP beginning from the heme-like binding pose observed in the wtBlc-split:heme co-crystal structure. Trajectories were featurized as all pairwise heavy atom distances between HP and wtBlc atoms within 4.0 Å of any HP atom in the initial heme-like binding pose. A lag time of 10 ps was used for time-lagged independent component analysis.

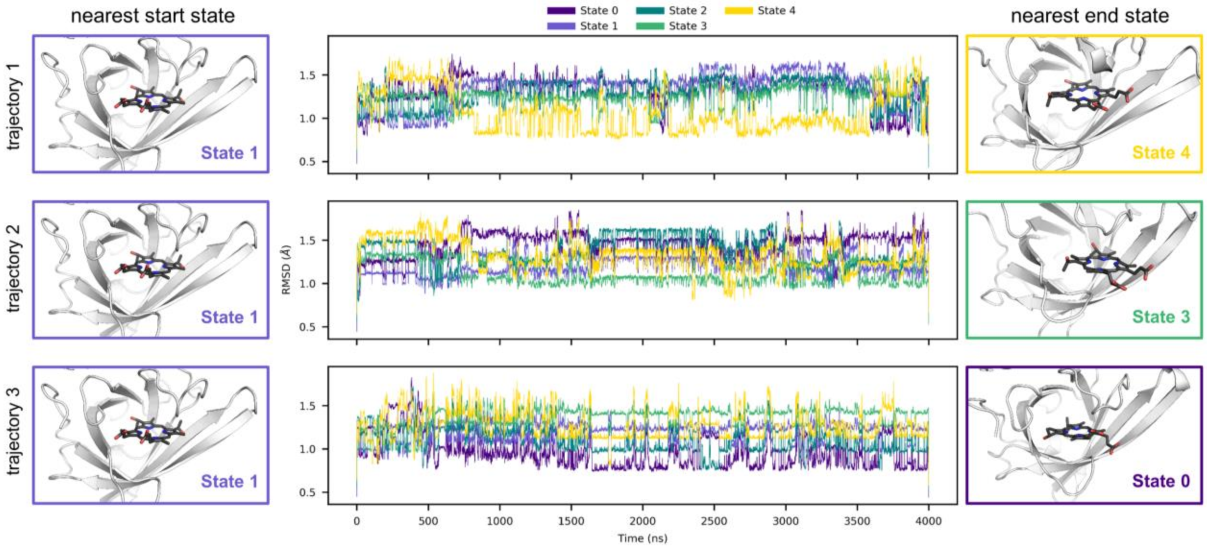

Figure S8. HP transitions from the initial heme-like binding mode to an alternative stable pose**.** All simulations trajectories began from the heme-like binding mode (left panel; HMM state 1; purple outline). Three independent simulations were run for 4.0 μs, and rmsd vs. time plots of HP for each of the HMM HP state binding modes were generated (middle panel). Visualization of the nearest HMM state pose to the last frame in each trajectory (right panel).

Table S2. Binding free energy estimates of wtBlc:HP in different HMM metastable states. A HMM distinguishing 5 metastable states was generated from 3 independent 4.0 μs MD simulations of WtBlc:HP. For each state, MM-PBSA was performed on 1000 randomly resampled trajectory frames. The IE was computed as a post-hoc analysis of MM-PBSA energies from the gas phase (electrostatic and Van der Waals) contributions to the interaction energy between the protein and ligand, as described by Duan et al.[1] Error represents standard error of the mean (SEM) across the 1000 frames used to compute the binding free energies.

Hidden Markov Model		MM-PBSA-IE (kcal/mol)						
State	Stationary Distribution (π)	E_{MM}	ΔG_{solv}	$-T\Delta S_{gas}$ (IE)	ΔG_{bind} (neglecting IE)	$\pi\Delta G_{bind}$ (neglecting IE)	ΔG_{bind} (including IE)	$\pi\Delta G_{bind}$ (including IE)
0	0.32	-144.65 ± 0.42	77.64 ± 0.30	42.69	-67.01	-21.44	-24.32	-7.78
1	0.16	-150.20 ± 0.43	77.58 ± 0.29	47.22	-72.62	-11.62	-25.40	-4.06
2	0.02	-140.48 ± 0.41	74.66 ± 0.26	34.43	-65.82	-1.32	-31.39	-0.63
3	0.22	-134.83 ± 0.31	68.78 ± 0.22	29.81	-66.05	-14.53	-36.24	-7.97
4	0.28	-150.66 ± 0.46	76.47 ± 0.30	42.85	-74.19	-20.77	-31.34	-8.78

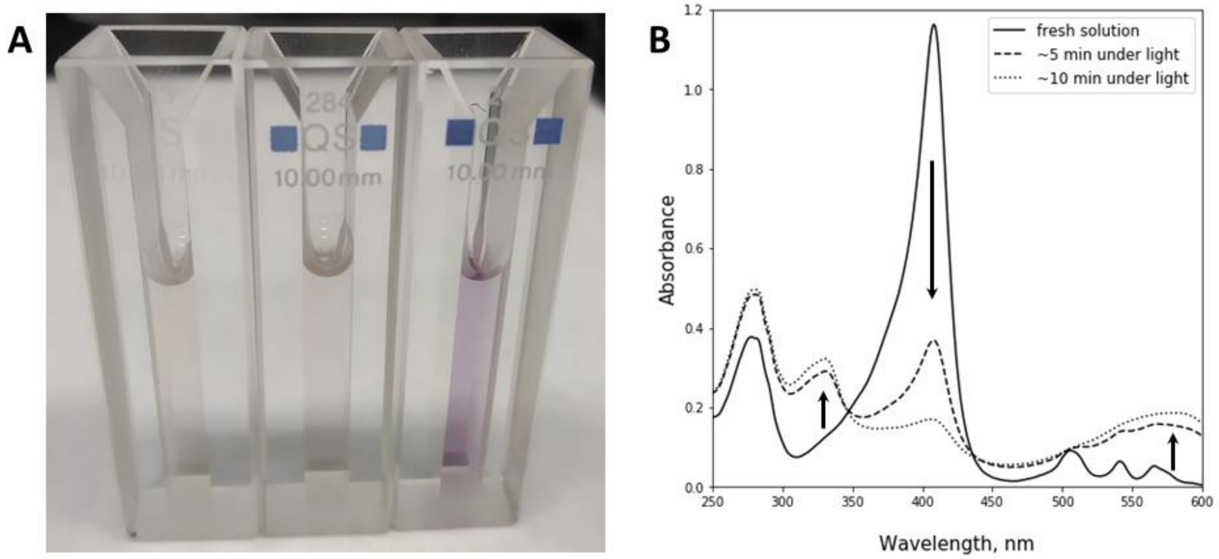

Figure S9. Light-induced changes of the wtBlc:HP solution. (A) Visible color change of the solution. Left to right: HP solution in buffer, fresh wtBlc:HP mixture, and wtBlc:HP mixture after 5 min under light. (B) Absorbance spectra of the fresh wtBlc:HP solution (black solid line), the wtBlc:HP solution after 5 min under light (black dashed line), and the wtBlc:HP solution after 10 min under light (black dotted line). Arrows show the directions of main spectral changes.

References

1. Duan, L., Liu, X. & Zhang, J.Z.H. Interaction Entropy: A New Paradigm for Highly Efficient and Reliable Computation of Protein–Ligand Binding Free Energy. *Journal of the American Chemical Society* **138**, 5722-5728 (2016).
